# Supplementary material for: The disintegration of event files over time: Decay or interference?
Source: Psychon Bull Rev. 2020 May 6;27(4):751–7. doi: 10.3758/s13423-020-01738-3 (PMC7399672; doi:10.3758/s13423-020-01738-3)
Supplement: Supplementary file 1 — (DOCX 42 kb) [file 13423_2020_1738_MOESM1_ESM.docx]

# The disintegration of event files over time:

# Decay or interference?

## Bernhard Hommel^1,2^ & Christian Frings^3^

^1^ Leiden University

Institute for Psychological Research & Leiden Institute for Brain and Cognition

Leiden, The Netherlands

^2^ Department of Psychology, Shandong Normal University, Jinan, China

^3^ Trier University

Institute for Psychology, Department of Cognitive Psychology,

Trier, Germany

Corresponding Address:

Bernhard Hommel

Leiden University

Department of Psychology

Cognitive Psychology Unit

Wassenaarseweg 52

2333 AK Leiden, The Netherlands

[bh@bhommel.onmicrosoft.com](mailto:memelink@fsw.leidenuniv.nl)

# Abstract

When facing particular combinations of stimuli and responses, people create temporary event files integrating the corresponding stimulus and response features. Repeating one or more of these features retrieves the entire event file, which impairs performance if not all features repeat (partial-repetition costs). We studied how durable event files are over time and how sensitive they are to intervening objects or stimulus-response events. After-effects of relevant and irrelevant stimulus-response bindings were assessed after intervals of 1 to 5 s between creation and retrieval of the binding that were either unfilled (Experiment 1A), filled with 0, 2, or 4 presentations of the same neutral stimulus (1B), or of changing stimuli (1C), or filled with 0, 2, or 4 task-unrelated stimulus-response combinations (2A) or the same number of repetitions of the binding-inducing stimulus-response combination (2B). Taken altogether, the findings show a strong impact on the duration of the interval but no systematic effect of the type and number of intervening events. This suggests that event files disintegrate over time, as a function of spontaneous decay, but not due to interference from other bindings.

The human brain codes external events in a distributed, feature-based fashion, which is true for perceptual modalities like vision (e.g., DeYoe & Van Essen, 1988) as well as for action (e.g., Georgopoulos, 1990). This raises the question of how and according to which rules features belonging to the same stimulus, response, or stimulus-response event are integrated. With regard to perception, Treisman and colleagues (Kahneman, Treisman, & Gibbs, 1992; Treisman, 1996) have suggested that the codes representing the features of the same object are temporarily integrated into what they call “object files”. In support of this assumption, they demonstrated that participants respond faster and more accurately if the identity of a visual object is repeated, but only in conditions that retain the relation between identity and location—suggesting that the previous encounter has left an identity-location binding behind.

Hommel (1998, 2004) has extended the object-file concept to actions and stimulus-response events, and suggested that their features are integrated into “event files”. Applying the logic of Treisman and colleagues, he was able to show that if participants are facing sequences of stimulus-response combinations, performance is better if the present stimulus and response features are either identical with the previous stimulus-response combination or if no feature is shared. In other words, performance is impaired if a stimulus feature repeats while the response changes, or vice versa—the *partial-*(feature-)*repetition cost*. This suggests that repeating a feature of a stimulus or response tends to retrieve previously created bindings involving that feature, which would be problematic if the other component(s) of the retrieved binding conflicts with one of the present features (see Henson, Eckstein, Waszak, Frings, & Horner, 2014; Frings et al., in press). Indeed, an fMRI study showed that repeating a stimulus feature activates the motor action that was previously paired with this feature and repeating an action activates the stimulus feature that was previously paired with this action (Kühn et al., 2011).

The main question we pursued in the present study was how durable event files are. Previous studies showed that event files can survive for at least 4 s between the first (binding-inducing) trial and the second (binding-retrieving) trial (Hommel & Colzato, 2004) in the case of target-response bindings while distractor-response bindings seem to survive for only about 2 s (Frings, 2011). However, these observations were made in the absence of any other stimulus or action between binding and retrieval, and therefore provide only a measure of pure memory decay over a relatively short period of time. Some more evidence was provided by Pösse, Waszak, and Hommel (2006), who found small but significant after-effects of bindings created three trials ago and even after a task switch. Frings and Rothermund (2011) also found distractor-response binding effects intact with one trial intervening between the binding-inducing and binding-retrieving trial. Yet, in the few previous studies timing and intervening trials were not systematically varied. Given that memory researchers disagree with respect to the question whether forgetting reflects pure decay or interference for more than 80 years (McGeoch, 1932; Altmann & Schunn, 2012), we thus aimed to disentangle decay and interference in a more systematic fashion. We did so by means of two experiments. In both experiments, we investigated two kinds of stimulus-response bindings: target-response binding (binding between relevant stimulus feature and response; Hommel, 1998) and distractor-response binding (binding between irrelevant stimulus feature and response; Hommel, 1998; Frings, Rothermund, & Wentura, 2007).

# Experiment 1A-C

Experiment 1 investigated whether event files are sensitive to the processing of other stimulus events (and the resulting object files) occurring between formation and retrieval of the respective binding. We compared the impact of unfilled intervals from 1 to 5 s between the trial that induced the creation of the stimulus-response bindings and the trial that induced the retrieval of these bindings (Experiment 1A), the impact of intervals that were filled with repeated presentation of the same neutral stimulus (1B), and the impact of intervals with changing stimuli (1C).

## Method

### Participants

Sixty adults took part for pay in single sessions, 20 each in Experiment 1A, 1B, 1C. All participants reported having normal or corrected to normal vision and were not familiar with the purpose of the experiment.

### Apparatus and stimuli

The experiment was controlled by a Hewlett Packard Vectra QS20 computer, attached to an Eizo 9080i (16-inch) monitor. Participants faced three grey square outlines on a black background, vertically arranged as illustrated in Fig. 1. From a viewing distance of about 60 cm, each square measured 1.2 x 1.2 deg. The uppercase letters O and X (0.3 x 0.4 deg) served as S1 and S2 alternatives, which were presented in white in the top or bottom frame. Intervening letters (fillers) appeared in the middle frame. Response cues also appeared in the middle frame (see Fig. 1), with rows of three white left- or right-pointing arrows indicating a left and right keypress (R1), respectively. Responses were made with the index fingers of the left or right hand, by pressing a left or right of two board-mounted microswitches, respectively.

### Procedure and design

Participants made two responses in each trial: A precued, simple R_1_ to S_1_ onset and a binary-choice R_2_ to S_2_ shape. R_1_ was a simple reaction with the left or right key, as indicated by the response cue, to be carried out as soon as the first stimulus (S_1_) appeared, independent of its shape or location. Participants were told that there would be no systematic relationship between S_1_ and R_1_ and they were encouraged to respond to the onset of S_1_ only, thus disregarding the stimulus attributes.

Between S_1_ and S_2_ there was an SOA of 1 s plus 0, 2, or 4 "filler intervals" of 1 s each. In Experiment 1A, these intervals were always unfilled (*No Filler*), that is, after the response cue only S_1_ and S_2_ appeared with an SOA of 1, 3, or 5 s. In Experiment 1B, the intervals were filled with the neutral uppercase letter I (*Repeated Filler*). That is, if S_1_ and S_2_ were separated by two or four 1-s intervals, the letter I was presented during the first 400 ms of each of these intervals—while the zero-interval condition was exactly as in Experiment 1A (i.e., no filler appeared). Participants were instructed to wait after their first response until an O or X would appear and then react according to the specified mapping rule, while the letter I should be ignored. In Experiment 1C, the intervening intervals were filled with random selections (without replacement) from the neutral letters I, J, L, and T (*Changing Filler*). That is, if S_1_ and S_2_ were separated by two or four intervals, a different neutral letter appeared in the first 400 ms of each of them. Participants were again to wait after their first response for another O or X and then react according to the specified mapping rule. R_2_ was a binary-choice reaction to the form of S_2_. Half of the participants responded to the O and X by pressing the left and right key, respectively, while the other half received the opposite mapping.

The sequence of events in each trial is illustrated in Fig. 1. Subsequent to the intertrial interval of 2000 ms, a response cue signaled R_1_ for 1500 ms, followed by a blank interval of 1000 ms. Then S_1_ appeared for 400 ms, followed by a blank interval of 600 ms and 0, 2, or 4 intervals of 1000 ms each, so that the SOA between the two target stimuli amounted to 1, 3, or 5 s. If R_1_ was incorrect or not given within 600 ms, a new trial was initiated. Otherwise S_2_ appeared—after the filler intervals, if applicable—and stayed until R_2_ was given or 2000 ms had passed. If any response was incorrect or missing, a short beep was presented, and the trial was recorded and repeated at some random position in the remainder of the block.

Each experiment consisted of 40 randomly determined practice trials and three experimental blocks. Each block comprised 96 randomly ordered trials, which were composed by a factorial combination of the two shapes and two locations of S_2_, the two possible relationships between S_1_ and S_2_ (i.e., repetition vs. alternation) regarding shape and location, the two possible relationships between R_1_ and R_2_ (repetition vs. alternation), and the three different numbers of filler intervals. A short break was allowed after each experimental block.


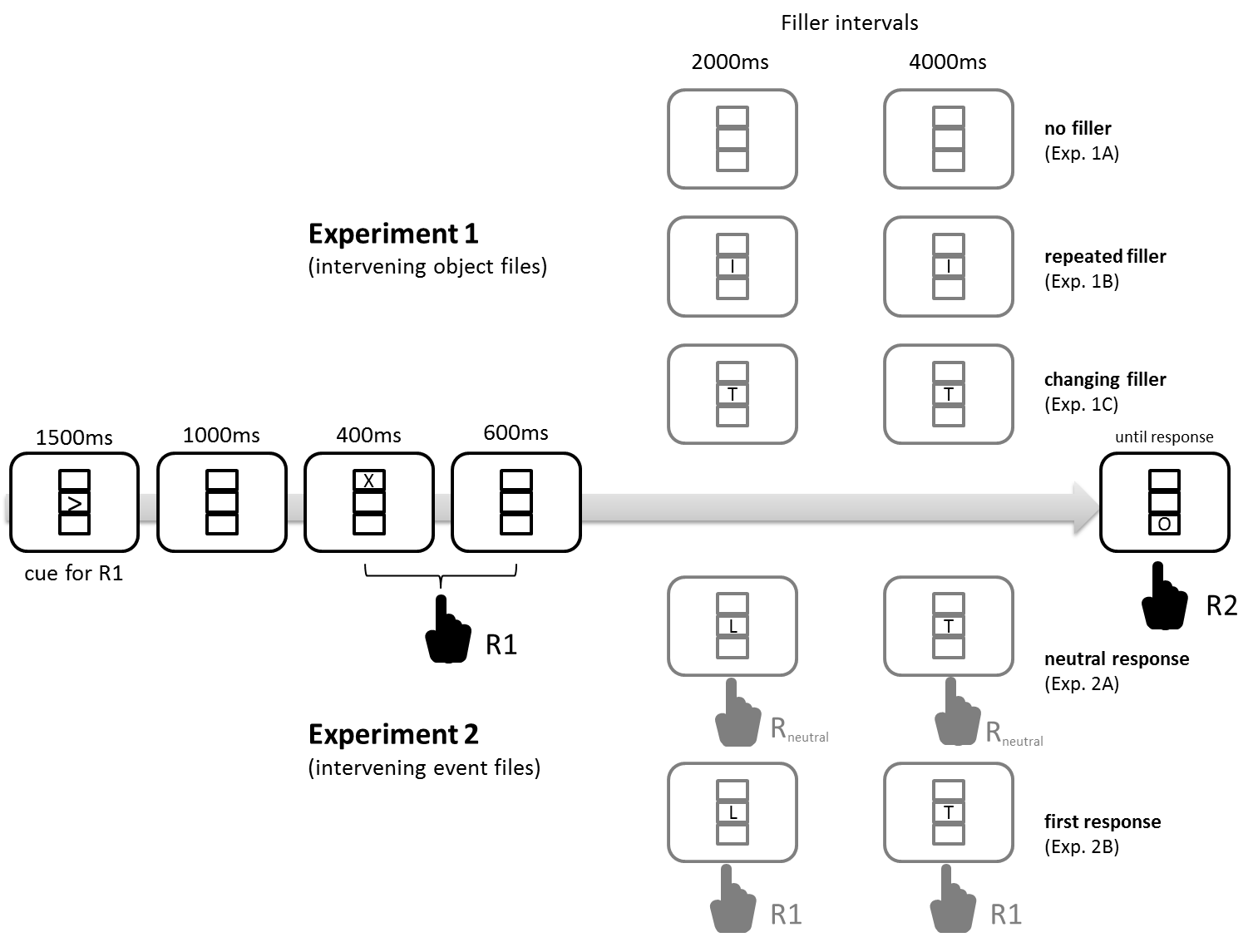


**Fig. 1** Trial sequence for Experiments 1 and 2. The dependent measures were taken from the shape classification response to the final display (R2). In Experiment 1, no filler, a repeating filler, or a changing filler was presented but participants did not respond to them. In Experiment 2, a changing filler appeared, and participants responded to it with a neutral response or by repeating R1. See text for further explanations. Stimuli are not drawn to scale

## Results

R1 was missing in 3.9% of all trials and was incorrect in 1.3% of the remaining in-time trials. Correct responses were given in 315 ms on average. Data for R1 were not further analyzed. Trials with a missing R2 (0.1% of all trials with a correct R1) were also excluded from analysis. As reported in the Supplement, Reaction times (RTs) and errors were analyzed by means of a Response Repetition x Location Repetition x Shape Repetition x SOA (1, 3, or 5 s) x Experiment (no filler vs. repeated filler vs. changing filler) MANOVA with Pillai’s trace as the criterion. However, for the sake of readability, we here report only results from three-way analyses of the S-R binding effects (i.e., the interactions of response repetition with the repetition of the task-relevant stimulus shape and with the repetition of the task-irrelevant stimulus location) as a function of SOA and Experiment (see Fig. 2).

### Binding of (task-relevant) stimulus shape and response

In RTs, the main effect of SOA was significant, *F*(2,56) = 23.97, *p* < .001, η_p_^2^ = .46, showing that the binding effects significantly decreased over time. Neither the main effect of Experiment, *F*(2,57) = 1.53, *p* = .22, η_p_^2^ = .05, nor the SOA-by-Experiment interaction, *F*(4,114) = 1.66, *p* = .16, η_p_^2^ = .06, was significant, suggesting that the decrease of the binding after-effect over time was not modulated by filler type. The error rates yielded the same pattern: while the main effect of SOA was significant, *F*(2,56) = 23.97, *p* < .001, η_p_^2^ = .55, the main effect of Experiment, *F*(2,57) = 1.54, *p* = .23, η_p_^2^ = .05, and the interaction, *F*(4,114) = 1.89, *p* = .16, η_p_^2^ = .05, were not.

### Binding of (task-irrelevant) stimulus location and response

In RTs, the main effect of SOA was significant, *F*(2,56) = 12.77, *p*<.001, η_p_^2^ = .31, showing that the binding effects significantly decreased over time. Again, neither the main effect of Experiment, *F*(2,57) = 1.15, *p* = .32, η_p_^2^ = .04, nor the interaction, *F*(4,114) = 2.06, *p* = .090, η_p_^2^ = .07, was significant, suggesting that the decrease of the binding after-effect over time was not modulated by filler type. The error rates yielded a comparable pattern, the main effect of SOA was significant, *F*(2,56) = 10.52, *p* < .001, η_p_^2^ = .27, but the main effect of Experiment was not, *F*(2,57) = 1.54, *p* = .23, η_p_^2^ = .05. This time the interaction effect was significant, *F*(4,114) = 2.77, *p* = .031, η_p_^2^ = .09, showing that the SOA effect varied with filler type. However, Fig. 2 indicates that this does not reflect a systematic pattern: the error functions compared well with those obtained for shape-response binding with respect to the shortest and the longest SOA; only the medium SOA showed a somewhat stronger drop of effect size from the first to the second SOA for the no-filler as compared to the changing-filler condition.


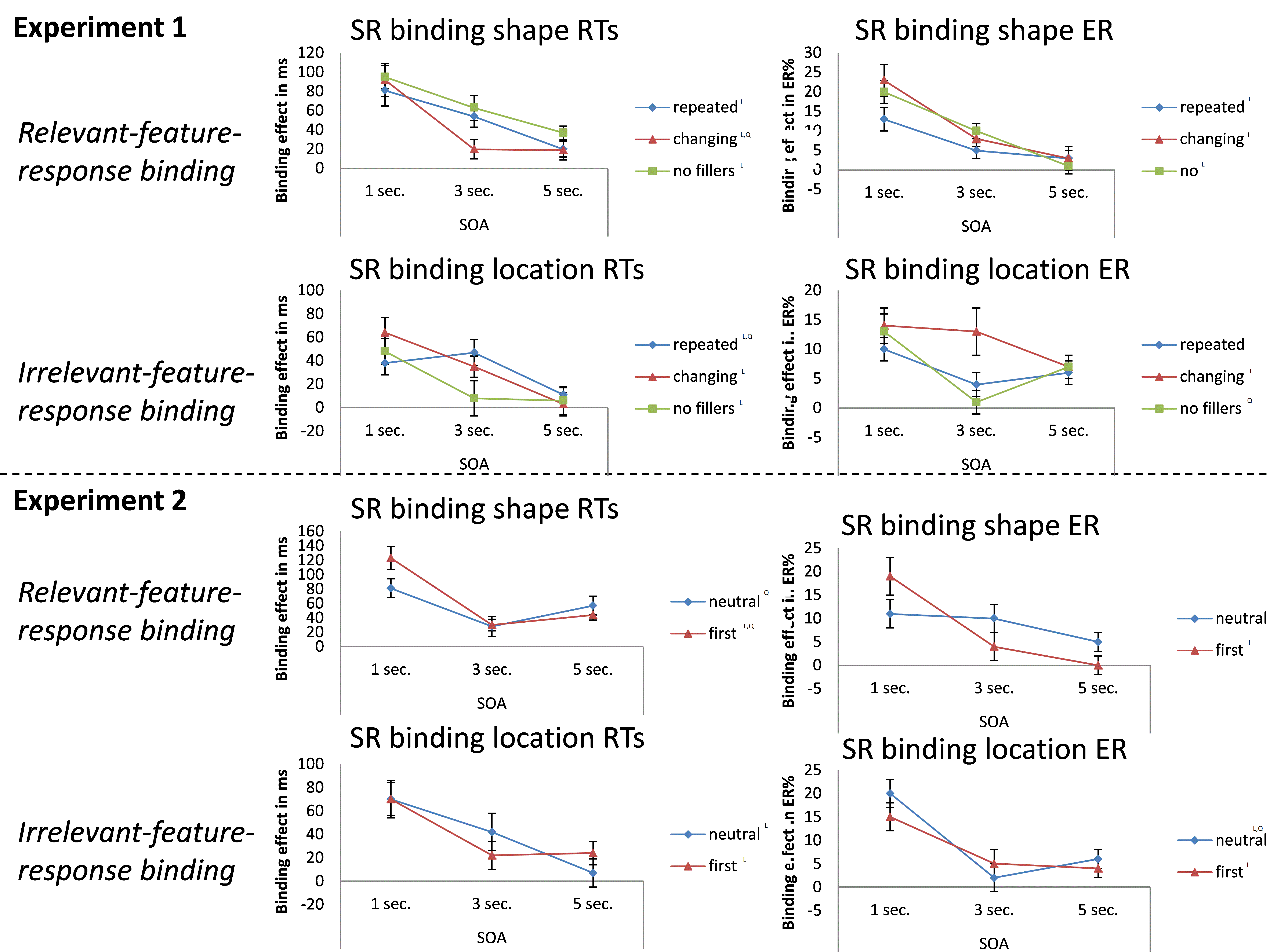


**Fig 2.** Shape-response and location-response binding effects (signed difference between alternation and repetition of a stimulus or response feature; e.g., RT_alternation_-RT_repetition_) in RT (ms) and error rates (%) for Experiment 1 and Experiment 2 as a function of SOA and experimental condition (filler type). L and Q as indices indicate significant (*p* < .05) linear and quadratic trends of the SOA/forgetting function of the corresponding condition, respectively

# Experiment 2A-B

Experiment 2 investigated whether event files are sensitive to the processing of other stimulus-response events (and the resulting event files) occurring between formation and retrieval of the respective binding. It did so by comparing the impact of intervals filled with task-unrelated stimulus-response combinations (2A) with the impact of intervals filled with repetitions of the response inducing the binding to new stimuli (2B). The key question was whether the binding effects would decay over time and/or whether the decay would depend on or be boosted by particular intervening events. Note that our design allowed us to assess effects of intervening events on task-relevant and task-irrelevant stimulus and stimulus-response bindings. Given that task-irrelevant bindings are known to be weaker than task-irrelevant bindings (Hommel, 1998)—presumably due to less effective retrieval of the former (Hommel, in press), it seems possible that the former are more strongly affected by intervening events than the latter.

## Method

Forty adults took part for pay in single sessions, 20 in Experiment 2A and 20 in 2B. All participants reported having normal or corrected‑to‑normal vision and were not familiar with the purpose of the experiment. The method was as in Experiment 1C (Changing Filler) with the following exceptions. Participants operated left and right response key with the index and middle finger on their dominant hand. In Experiment 2A each neutral letter was to be responded to by pressing the space bar of the computer keyboard (operated by the nondominant hand), that is, one press for each neutral letter. Experiment 2B also required a response to each of the neutral letters but here participants were to repeat their first reaction. That is, they performed R1 in response to S1 and repeated that response with each new letter until S2, the second O or X, signaled R2.

## Results

R_1_ was missing in 2.9% of all trials and was incorrect in 0.5% of the remaining in-time trials. Correct R_1_ responses were given in 279 ms on average. After excluding trials with a missing R_2_ (0.09% of all trials with a correct R_1_) R_2_ data were analyzed analogously to Experiment 1. The analytical approach was exactly as in Experiment 1, except that the Experiment factor had only two levels (neutral response vs. R1 repetition). Results are shown in Fig. 2.

### Binding of (task-relevant) stimulus shape and response

In RTs, the main effect of SOA was significant, *F*(2,37) = 21.27, *p* < .001, η_p_^2^ = .54, showing that the after-effects of binding decreased over time. The main effect of Experiment was again not significant, *F*(1,38) = 0.74, *p* = .397, η_p_^2^ = .02, but the interaction of SOA and Experiment approached the significance criterion, *F*(2,374) = 3.23, *p* = .051, η_p_^2^ = .15. A closer look reveals that this was due to a numerically stronger effect of filler type at the first SOA, which disappeared at the longer SOAs. The error rates yielded the same pattern, with a significant main effect of SOA, *F*(2,37) = 9.20, *p* = .001, η_p_^2^ = .33, a non-significant main effect of Experiment, *F*(1,38) = 0.14, *p* = .714, η_p_^2^<.01, and a significant interaction, *F*(2,37) = 3.91, *p* = .029, η_p_^2^ = .18. Again, the reason was a stronger effect of filler type at the first as compared to the other SOAs.

### Binding of (task-irrelevant) stimulus location and response

In RTs, the main effect of SOA was significant, *F*(2,37) = 7.21, *p* = .002, η_p_^2^ = .28, showing that the binding effects significantly decreased over time. Neither the main effect of Experiment, *F*(1,38) < 0.01, *p* = .935, η_p_^2^ < .01, nor the interaction, *F*(2,37) = 1.01, *p* = .373, η_p_^2^ = .05, was significant, suggesting that the decrease of the binding after-effect over time was not modulated by filler type. The error rates yielded the same pattern: while the main effect of SOA was significant, *F*(2,37) = 25.99, *p* < .001, η_p_^2^ = .58, the main effect of Experiment, *F*(2,57) = 1.54, *p* = .23, η_p_^2^ = .05, and the interaction, *F*(2,37) = 1.15, *p* = .327, η_p_^2^ = .06, were not.

# General discussion

The aim of this study was to compare the impact of temporal decay and interference by other object or event files on the after-effects of stimulus-response binding. Two observations are of particular importance for assessing the impact of time and interference.

First, all eight analyses of target-response and distractor-response binding effects on RTs and error rates showed a clear SOA effect, suggesting that the after-effects of stimulus-response bindings disappear over time. On the one hand, the time it took these after-effects to approach the lower asymptote (about 5 s) was relatively short, suggesting that event files are more related to short- than long-term memory. On the other hand, however, it is important to consider that our study, and event-file studies in general, keep repeating and alternating very few stimulus and response features over many trials. If an event file is created in every trial, this must generate substantial amounts of noise produced by still somewhat active event files that are frequently reactivated by a reoccurring stimulus or response features. This might differ from conditions in which each event file is unique by not sharing any features with other event files. Hence, even our pure decay condition in Experiment 1A might be considered to have included a certain degree of proactive interference from lingering event-file activation. Whether this did or did not play a role, our findings suggest that event files can become functionally disintegrated after about 5 s.

Second, five out of eight analyses did not provide any evidence for a modulation of this disintegration through intervening stimulus or stimulus-response events. This suggests that intervening events did not contribute to the disintegration, which implies that the number and type of recently created object or event files does not induce any competition with other event files, at least not any more competition than the general “noise” discussed above might generate. Hence, there does not seem to be a strict capacity limitation regarding the concurrent maintenance of object or event files. It is true that the three remaining analyses did produce interactions between condition and SOA that reached or were close to the significance criterion. However, a closer look revealed that the causes responsible for these interactions (Experiment 1: stronger drop from 1st to 2nd SOA in one condition with later convergence of all conditions at the 3rd SOA; Experiment 2: difference of the two conditions at the 1st SOA that disappeared and later SOAs) are unlikely to do with different decay rates. True, the inconsistent pattern of the interaction effects of intervening events and binding is harder to interpret, which is why we hesitate to conclude that there is no interference at all. Yet, Bayesian statistics and post hoc power analyses^[[1]](#footnote-1)^ suggest that interference effects are unlikely to be of a size that could fully account for the observed decrease of binding effects over time. Taken together, it is fair to conclude that the main impact on binding effects is due to decay not interference.

Finally, a closer look at the SOA/forgetting functions of binding effects suggests that the observed decay functions is linear (indeed, linear trends do fit the data quite well), which might be taken to be at odds with the common findings of power functions in memory research. Yet, given the evidence that linear and power functions can be converted into each other (Kahana & Adler, 2002), and that we have only three SOA levels for measuring trends here, we hesitate to put much emphasis on this observation. Future research might more effectively investigate the question whether event-file decay differs from the typical memory power function by using more fine-grained SOA levels.

Our findings suggest that longer temporal distances between the creation and the retrieval of stimulus-response bindings tend to reduce their impact, suggesting that successful retrieval becomes less likely as a function of time. And yet, whether anything happens during that time does not seem to play any role for the reduced impact. In particular, the creation of other object or event files during that time does not seem to create any competition or interference that would make the retrieval of other files less likely. Hence, we did not find direct evidence for interference, even though it remains possible that (proactive) interference plays a somewhat more indirect role by increasing internal noise that in turn boosts decay. This apparent lack of capacity limitations for event files must be considered particularly remarkable as responses to intervening events (Spadaro & Milliken, 2012) and even entirely irrelevant stimulus events (as evidenced by the suffix effect, Crowder, 1967) have been shown to interfere with performance in more conventional short-term memory paradigms.

Taken altogether, our findings suggest that the after-effects of stimulus-response binding lose impact over (relatively short) time, independently from other intervening events. We note, however, that this conclusion only holds for short-term bindings and trial-to-trial effects, as investigated in the present study. It may well be that bindings can be transformed into longer lasting S-R episodes (Moeller & Frings, 2017; Frings, Moeller, & Horner, 2017), the retrieval of which might be governed by other principles.

# Open Practices Statement

Aggregated data for this article can be found online at [www.osf.io/kjtfx](http://www.osf.io/kjtfx).

# References

Altmann, E.M., & Schunn, C.D. (2012). Decay versus interference: A new look at an old interaction. *Psychological Science, 23*, 1435–1437.

Crowder, R.G. (1967). Prefix effects in immediate memory. *Canadian Journal of Experimental Psychology, 21*, 450–461.

DeYoe, E. A., & Van Essen, D. C. (1988). Concurrent processing streams in monkey visual cortex. *Trends in Neuroscience, 11*, 219–226.

Faul, F., Erdfelder, E., Lang, A., & Buchner, A. (2007). GPower 3: A flexible statistical power analysis program for the social, behavioral and biomedical sciences. *Behavioral Research Methods, 39*, 175–191.

Frings, C. (2011). On the decay of distractor-response episodes. *Experimental Psychology*, *58*, 125–131.

Frings, C., Moeller, B., & Horner, A. (2015). On the durability of bindings between responses and response-irrelevant stimuli. *Acta Psychologica*, *161*, 73–78.

Frings, C., Rothermund, K. & Wentura, D. (2007). Distractor repetitions retrieve previous responses to targets. *Quarterly Journal of Experimental Psychology*, *60*, 1367–1377.

Frings, C., Koch, I., Rothermund, K., Dignath, D., Giesen, C., Hommel, B., *et al.* (in press). Merkmalsintegration und Abruf als zentrale Prozesse der Handlungssteuerung – eine Paradigmen-übergreifende Perspektive [Feature binding and retrieval as central processes of action control – an across-paradigm perspective]. *Psychologische Rundschau*.

Frings, C., & Rothermund, K. (2011). To be or not to be...included in an event file: Integration and retrieval of distractors in stimulus-response episodes is influenced by perceptual grouping. *Journal of Experimental Psychology: Learning, Memory, & Cognition*, *37*, 1209–1227.

Georgopoulos, A. P. (1990). Neurophysiology of reaching. In M. Jeannerod (Ed.), *Attention and Performance XIII: Motor representation and control* (pp. 227–263). Hillsdale, NJ: Erlbaum.

Henson, R. N., Eckstein, D., Waszak, F., Frings, C., & Horner, A. J. (2014). Stimulus-response bindings in priming. *Trends in Cognitive Sciences*, *18*, 376–384.

Hommel, B. (1998). Event files: Evidence for automatic integration of stimulus-response episodes. *Visual Cognition, 5*, 183–216.

Hommel, B. (2004). Event files: Feature binding in and across perception and action. *Trends in Cognitive Sciences, 8*, 494–500*.*

Hommel, B. (in press). Theory of Event Coding (TEC) V2.0: Representing and controlling perception and action. *Attention, Perception, & Psychophysics*.

Hommel, B., & Colzato, L. S. (2004). Visual attention and the temporal dynamics of feature integration. *Visual Cognition, 11*, 483–521.

Kahana, M. J., & Adler, M. (2002). Note on the power law of forgetting. *bioRxiv* 173765; doi: https://doi.org/10.1101/173765

Kahneman, D., Treisman, A., & Gibbs, B. J. (1992). The reviewing of object files: Object-specific integration of information. *Cognitive Psychology, 24*, 175–219.

Kühn, S., Keizer, A., Colzato, L.S., Rombouts, S.A.R.B., & Hommel, B. (2011). The neural underpinnings of event-file management: Evidence for stimulus-induced activation of, and competition among stimulus-response bindings. *Journal of Cognitive Neuroscience, 23*, 896–904.

McGeoch, J. A. (1932). Forgetting and the law of disuse. *Psychological Review, 39*, 352–370.

Moeller, B. & Frings, C. (2017). Overlearned responses hinder S-R binding. *Journal of Experimental Psychology: Human Perception and Performance*, *43*, 1–5.

Pösse, B., Waszak, F., & Hommel, B. (2006). Do stimulus-response bindings survive a task switch? *European Journal of Cognitive Psychology, 18*, 640–651.

Treisman, A. (1996). The binding problem. *Current Opinion in Neurobiology, 6*, 171–178.

Wagenmakers, E.–J., Wetzels, R., Borsboom, D., & van der Maas, H. L. (2011). Why psychologists must change the way they analyze their data: The case of psi: Comment on Bem (2011). *Journal of Personality and Social Psychology, 100*, 426–432. doi:10.1037/a0022790

# Supplement

**Table S1** Full MANOVA model for RTs in Experiment 1 (A–C). The RTs were submitted to a Response Repetition (RR) x Location Repetition (L) x Shape Repetition (S) x SOA (1 s vs. 3 s vs. 5 s) x Experiment (no filler vs. repeated filler vs. changing filler) MANOVA with Pillai’s trace as the criterion

| Effect | F-value | *p* value | Partial eta^2^ |
| --- | --- | --- | --- |
| RR | 5.08 | .028 | .08 |
| L | 39.01 | < .001 | .41 |
| S | 3.35 | .072 | .06 |
| SOA | 30.43 | < .001 | .52 |
| Exp | 0.81 | .451 | .03 |
| RRxL | 56.84 | < .001 | .50 |
| RRxS | 113.41 | < .001 | .67 |
| RRxSOA | 1.83 | .170 | .06 |
| RRxExp | 0.83 | .439 | .03 |
| LxS | 16.72 | < .001 | .23 |
| LxSOA | 0.97 | .385 | .03 |
| LxExp | 3.20 | .048 | .10 |
| SxSOA | 5.41 | .007 | .16 |
| SxExp | 2.60 | .083 | .08 |
| SOAxExp | 3.84 | .006 | .12 |
| RRxLxS | 8.21 | .006 | .13 |
| RRxLxSOA | 12.77 | < .001 | .31 |
| RRxLxEXP | 1.15 | .324 | .04 |
| RRxSxSOA | 23.97 | < .001 | .46 |
| RRxSxExp | 1.54 | .224 | .05 |
| RRxSOAxExp | 1.04 | .389 | .04 |
| LxSxSOA | 0.76 | .474 | .03 |
| LxSxExp | 0.63 | .538 | .02 |
| LxSOAxExp | 1.43 | .229 | .05 |
| SxSOAxEXP | 0.67 | .614 | .02 |
| RRxLxSxSOA | 0.38 | .688 | .01 |
| RRxLxSxExp | 1.44 | .246 | .05 |
| RRxLxSOAxExp | 2.06 | .090 | .07 |
| RRxSxSOAxExp | 1.66 | .164 | .06 |
| LxSxSOAxExp | 0.86 | .492 | .03 |
| RRxLxSxSOAxExp | 0.82 | .516 | .03 |

*Note*. The RRxLxSOA interaction corresponds to the main effect of SOA in Experiment 1 when binding effects are the DV. In the same vein, the RRxLxSOAxExp interaction corresponds to the two-way interaction of SOA x Experiment with binding effects as the DV in Experiment 1. The same logic applies to shape.

**Table S2** Mean RTs and error percentage in brackets in Experiment 1 (A–C) as a function of Response Repetition (RR) x Location Repetition (L) x Shape Repetition (S) x SOA (1 s vs. 3 s vs. 5 s) x Experiment (no filler vs. repeated filler vs. changing filler)

|  |  |  | **SOA 1 s** | | | **SOA 3 s** | | | **SOA 5 s** | | |
| --- | --- | --- | --- | --- | --- | --- | --- | --- | --- | --- | --- |
|  |  |  | *No filler* | *Repeated filler* | *Changing filler* | *No filler* | *Repeated filler* | *Changing filler* | *No filler* | *Repeated filler* | *Changing filler* |
| *RR* | *L rep.* | *S rep.* | 482 (3.0) | 473 (1.5) | 516 (3.1) | 478 (2.1) | 492 (1.9) | 491 (4.4) | 482 (2.2) | 444 (5.6) | 469 (2.6) |
|  |  | *S unr.* | 519 (6.6) | 501 (11.5) | 563 (6.5) | 512 (6.5) | 499 (6.2) | 519 (4.4) | 496 (2.4) | 458 (4.5) | 494 (3.2) |
|  | *L unr.* | *S rep.* | 508 (3.5) | 532 (4.8) | 555 (3.6) | 501 (3.3) | 518 (8.1) | 539 (6.4) | 483 (4.3) | 462 (7.1) | 497 (5.7) |
|  |  | *S unr.* | 544 (21.5) | 552 (21.7) | 584 (14.6) | 519 (8.8) | 526 (14.5) | 564 (8.7) | 509 (5.2) | 457 (8.7) | 512 (5.8) |
| *RC* | *L rep.* | *S rep.* | 554 (15.7) | 534 (17.1) | 577 (11.9) | 528 (8.7) | 516 (16.3) | 544 (9.9) | 503 (8.8) | 461 (8.9) | 503 (10.4) |
|  |  | *S unr.* | 506 (6.3) | 494 (8.2) | 542 (6.8) | 511 (3.4) | 519 (12.0) | 525 (5.3) | 495 (5.7) | 454 (7.2) | 506 (5.5) |
|  | *L unr.* | *S rep.* | 542 (10.6) | 553 (11.0) | 577 (7.2) | 554 (8.6) | 523 (9.1) | 552 (8.2) | 514 (2.2) | 475 (5.5) | 518 (4.9) |
|  |  | *S unr.* | 474 (1.2) | 457 (0.8) | 527 (1.2) | 498 (4.4) | 495 (7.5) | 515 (5.3) | 486 (4.0) | 452 (2.6) | 515 (4.8) |

*Note*. Repeated ~ rep.; unrepeated ~ unr. Response Repetition (RR), Response Change (RC)

**Table S3** Full MANOVA model for RTs in Experiment 2 (A–B). The RTs were submitted to a Response Repetition (RR) x Location Repetition (L) x Shape Repetition (S) x SOA (1 s vs. 3 s vs. 5 s) x Experiment (neutral vs. R1 repetition) MANOVA with Pillai’s trace as the criterion

| Effect | F-value | *p* value | Partial eta^2^ |
| --- | --- | --- | --- |
| RR | 0.64 | .431 | .02 |
| L | 0.37 | .547 | .01 |
| S | 4.18 | .048 | .10 |
| SOA | 25.59 | < .001 | .58 |
| Exp | 0.53 | .472 | .01 |
| RRxL | 52.02 | < .001 | .58 |
| RRxS | 94.41 | < .001 | .71 |
| RRxSOA | 9.28 | .001 | .33 |
| RRxExp | 4.20 | .048 | .10 |
| LxS | 10.54 | .002 | .22 |
| LxSOA | 10.57 | < .001 | .36 |
| LxExp | 0.52 | .476 | .01 |
| SxSOA | 20.76 | < .001 | .53 |
| SxExp | 0.54 | .467 | .01 |
| SOAxExp | 2.25 | .120 | .11 |
| RRxLxS | 10.79 | .002 | .22 |
| RRxLxSOA | 7.21 | .002 | .28 |
| RRxLxEXP | 0.01 | .935 | < .001 |
| RRxSxSOA | 21.27 | < .001 | .54 |
| RRxSxExp | 0.74 | .397 | .02 |
| RRxSOAxExp | 1.07 | .353 | .06 |
| LxSxSOA | 2.28 | .116 | .11 |
| LxSxExp | 1.46 | .235 | .04 |
| LxSOAxExp | 2.44 | .101 | .12 |
| SxSOAxEXP | 0.83 | .446 | .04 |
| RRxLxSxSOA | 1.74 | .190 | .09 |
| RRxLxSxExp | 0.23 | .637 | .01 |
| RRxLxSOAxExp | 1.01 | .373 | .05 |
| RRxSxSOAxExp | 3.23 | .051 | .15 |
| LxSxSOAxExp | 0.29 | .750 | .02 |
| RRxLxSxSOAxExp | 1.97 | .154 | .10 |

*Note*. The RRxLxSOA interaction corresponds to the main effect of SOA in Experiment 2 when binding effects are the DV. In the same vein, the RR x L x SOA x Exp interaction corresponds to the two-way interaction of SOA x Experiment with binding effects as the DV in Experiment 2. The same logic applies to shape.

**Table S4** Mean RTs and error percentage in brackets in Experiment 2 (A–B) as a function of Response Repetition (RR) x Location Repetition (L) x Shape Repetition (S) x SOA (1 s vs. 3 s vs. 5 s) x Experiment (neutral vs. first response)

|  |  |  | **SOA 1 s** | | **SOA 3 s** | | **SOA 5 s** | |
| --- | --- | --- | --- | --- | --- | --- | --- | --- |
|  |  |  | *Neutral* | *first* | *Neutral* | *first* | *Neutral* | *first* |
| *RR* | *L rep.* | *S rep.* | 515 (12.8) | 541 (2.2) | 533 (9.5) | 546 (4.8) | 462 (1.9) | 495 (1.9) |
|  |  | *S unr.* | 538 (16.4) | 568 (8.7) | 549 (11.3) | 569 (4.3) | 508 (5.1) | 512 (2.5) |
|  | *L unr.* | *S rep.* | 534 (27.4) | 568 (8.4) | 558 (12.1) | 553 (5.9) | 502 (4.7) | 509 (7.1) |
|  |  | *S unr.* | 545 (33.5) | 598 (23.5) | 576 (12.6) | 575 (11.0) | 534 (6.4) | 544 (3.7) |
| *RC* | *L rep.* | *S rep.* | 548 (9.5) | 611 (14.0) | 546 (14.4) | 583 (10.8) | 510 (10.0) | 535 (6.2) |
|  |  | *S unr.* | 507 (2.9) | 543 (5.6) | 561 (5.0) | 579 (8.3) | 490 (5.5) | 524 (3.7) |
|  | *L unr.* | *S rep.* | 516 (5.1) | 595 (8.9) | 555 (13.8) | 571 (8.6) | 533 (4.5) | 541 (4.7) |
|  |  | *S unr.* | 427 (0.0) | 474 (1.1) | 519 (4.8) | 560 (7.9) | 518 (4.2) | 516 (3.8) |

*Note*. Repeated ~ rep.; unrepeated ~ unr. Response Repetition (RR), Response Change (RC)

1. Post-hoc power analyses (computed with G-Power 3.1; Faul, Erdfelder, Buchnen, & Lang, 2007) suggest that the power to observe between-participants interaction effects of the same size as the decay effects was at least 1-ß = .92 in our study. In addition, Bayes factors for interference effects were between 0.6 and 4, suggesting indecisive evidence (Wagenmakers, Wetzels, Borsboom, & van der Maas, 2011). [↑](#footnote-ref-1)
